# Miscellaneous

**Published:** 1874-07

**Authors:** 


### MISCELLANEOUS.

### HEALTH RESORTS.

### THE LAKE COUNTRY.

Examine a well defined map of the State of New York and you will find a region embraced within the counties of Onondaga, Cayuga, Seneca, Yates and Ontario, known as the "Lake Country," which, for picturesque beauty and salubrity of climate, is unsurpassed by any other section of country. Here are those beautiful Lakes Skaneateles, Owasco, Cayuga, Seneca, Crooked and Canandaigua, running in a northerly and southerly direction, and nearly parallel with each other—from their position claiming the country for themselves against the encroachments of any great public thoroughfare from east to west. This fact, added to the unsurpassed beauty of this "Lake Country," will cause it to grow in favor as a summer resort for those in search of health and quiet recreation. Conceding all that can be asked by the admirers of the quiet beauty of these lakes and the country adjacent, we propose in this article to confine our attention to the Seneca—the largest, and, probably, most attractive of all—and the country around it. To be consistent, we should do so as a matter of course, since we are treating of "Health Resorts," and it is now generally conceded that mineral waters are among the most efficient remedies in the cure of many diseases. Whatever springs exist near the shores of other lakes mentioned, we are not aware that it is claimed there are any that rival the well known Magnetic and Sulphur Springs of Havana and Watkins, at the head of the Seneca Lake. It is a rule in medicine that remedies are always more effective when the mind of the patient is quieted, and his admiration aroused by pleasant surroundings. We believe, for a person, or a party of friends seeking health or recreation, or both, a few weeks could in no way be spent with more satisfaction or advantage than by following a course the outlines of which we will suggest. Leaving New York in the morning by the Erie Railway, which is the most direct, we arrive at Havana or Watkins in time for supper. Whether sick or well we should by all means stop at the "Magnetic Springs Sanitarium," which is a hotel near the Glen at Havana, or at the "Glen Park Hotel," which is connected with the Watkins Magnetic and Sulphur Springs, near the Glen at Watkins. Besides being first class hotels in all other respects, they possess the advantage of having an abundance of water in every room, brought from these flowing springs. As to location, we prefer them to any of the hotels of Havana or Watkins, for reasons which will be obvious to almost every visitor. When settled at the hotel, a visit to both the Watkins and Havana Glens will be in order. Then there are fine drives in every direction. One of the most beautiful is around the lake shore, on what is called the "Hector Falls" road. The trees form a complete arch over the road for miles. The ascent from Watkins is gradual. At a distance of three miles you are at an elevation of, perhaps, 200 feet above the lake, which, with the villages along the shore, seem spread out beneath you like a map. A very pleasant trip is to Cayuta Lake, about six miles from Havana. There is very good fishing in this lake. Fish are not abundant in the Seneca, on account of the low temperature of the water, it is said. There is good fishing in Crooked Lake, near Penn Yan, twenty-six miles from Watkins by railroad. There is also a very comfortable steamer running daily from Penn Yan to Hammondsport—sixteen miles and back. There are many ways of passing the time pleasantly and profitably in and around Watkins. But, perhaps, the crowning pleasure will be an occasional trip to Geneva and back, on one of the beautiful steamers that perform their daily trips summer and winter. The distance from Watkins to Geneva is forty miles. There are two departures from Watkins and Geneva daily. The time is only three hours, including landings. The boats are neatly fitted up, the cabins comfortable, and the fare excellent. He who has not breakfasted or dined on board one of these Seneca Lake steamers has a pleasure still in store for him.

### SAFETY OF ANÆSTHETICS.

If the force of statistics be of any value, ether appears, beyond question, to be the safest anæsthetic. By combining

American and British data relating to this question, the result shows conclusively that chloroform is eight times as dangerous as ether, twice as dangerous as a mixture of chloroform and ether, and, as far as experience goes, it is more dangerous than bichloride of methylene. The report of the London Chloroform Committee, appointed to investigate this subject, states that not only is ether less dangerous than chloroform, but that with every care and the most exact dilution of the chloroform vapor by the most skilful hands, the state of insensibility may pass in a few moments into one of imminent death.

### SYRUPS.

### ARE THEY ADULTERATED?

THERE is a syrup made from starch—a thick, heavy, whitish syrup, not as sweet as cane sugar syrup—which is sold for what it is by the manufacturers to the trade direct, or to manipulators of syrups, for mixing with low grade molasses or sugar syrups, to give them body and brightness. This syrup, properly made, is entirely unobjectionable, so far as adulteration is concerned, and in itself would be least likely to respond to the fallacious tea test so commonly recommended. This starch syrup may be made from corn, potatoes, or any other starch yielding vegetable substance. It is manufactured largely in Europe, and imported to this country under the name of glucose. We report it regularly each week among our importations and withdrawals from bond. It comes as a solid, also, as grape sugar, or starch sugar. It is used to a great extent by confectioners in manufacturing certain descriptions of their goods. There is nothing at all objectionable about it, and it depends entirely upon what is meant by adulteration whether syrups containing it are adulterated or not. If by adulteration is meant the act of mixing a hurtful, poisonous, or deleterious substance with another not so, then it is no adulteration to mix starch sugar syrup with cane sugar syrup. If by adulteration is meant the act of mixing a cheaper but harmless substance with a dearer one of similar characteristics, then such mixed syrups are adulterated. If sold unmixed, and upon its own merits, the starch or grape sugar syrup, properly made, is as free from adulteration as it is possible to manufacture any article. If sold for a regular cane sugar syrup a lie is told, and that is all there is about it. It is a possible thing to manufacture a starch syrup from old rags, sawdust, and other similar refuse, and the process is the same in theory as when the best corn is used, and the product may be made, with proper care, as pure as when seemingly less objectionable substances are used; but we do not know, of our own knowledge, of the existence of a single such factory in the United States.

In order to place the minds of our friends in the trade, who sell syrups, completely at ease, we called upon Professor Chandler, President of our Board of Health, to whom, it will be remembered, the Grand Jury referred the charge recently presented to it upon this subject, and who stands at the head of his profession as an analytical chemist. He informed us that the whole matter has been repeatedly and thoroughly examined by him, and that his report upon the reference above mentioned will be ready in a few days. We will give it in full at the earliest practicable moment, but in the meantime are permitted to state that out of a large number of samples of sugar and syrup, procured from a variety of sources, where, if ever, adultered articles would most likely be offered for sale, not one of the samples of sugar showed any adulteration, and but one of the samples of syrup. In that one he found .02 per cent. of chloride of tin—an amount so insignificant as to be absolutely more healthful than otherwise.

### AFTER A WHILE.

After a while a busy brain
Will rest from all its toil and pain.

After a while earth's rush will cease,
And a wearied heart find sweet release.

After a while a vanished face,
An empty seat, a vacant place.

After a while a man forgot,
A crumbled headstone, an unknown spot.

### "DUM SPIRO, SPERO."

A long, long lane—a bewildering lane—
Shrouded in darkness by moss-hung trees,
Whose limbs in a wild and weird refrain
Murmur their woes to the passing breeze,
Matted and meshed 'mid brier and thorn,
Wreathes and winds the tortuous way ;
Where toads and bats in a gloom forlorn,
Through the dark and dismal shadows stray.

And in all the air no beautiful thing—
Flowering blossom of dazzling hue—
Nor quivering insect on gay gauze wing—
Glistens in sunlight and falling dew.
And the light clouds flutter athwart the sky,
But never reflect in the pool below ;
And the redbreast shuns, as he passes by,
The lane where no beautiful thing can grow.

Yet, in wearisome travail, again and again—
Careless of thorns, and the jagged edge
Of the pitiless stones—unheeding the pain—
Must tired feet toil through brier and sedge ;
Must toil-worn limbs and sinking heart—
Passion-tortured, forsaken of men—
Still wearily wage a life apart
In the dreary lane—again and again.

But joy for the soul ! We can scoff at life,
With smiles for its sorrow, and scorn for its wrong ;
For the term of the wildest and bitterest strife,
Be it harsh as may be, will not be for long ;
And the longest, gloomiest lane there is—
Mocked by sweet memories of the past—
Must meet at its close. Oh, thought of bliss !
A turning to happier days at last.

Frank H. Norton.

### COLOR.

We do not study sufficiently the art of color; and by this neglect the effect of many an expensive wardrobe or elaborate piece of furniture is disagreeable. One or two colors should be dull, and not too pale; this is not generally known, or it is forgotten, and the result is the coarse and vulgar contrasts that we see around us. Blue is a favorite color, yet it is rare in nature; there are but few blue flowers; there is no blue in the human race; blue eyes many fancy they possess, but a clear blue is the rarest thing in nature. Green is becoming in itself, because it annuls any tinge of green which may be latent to the complexion. Deep, heavy reds are much used by the old Italian artists for drapery, but they need to be contrasted, as it would be difficult to do in dress. Yellow is an unjustly despised color; it has many beautiful shades, and only when too pure proves unmanageable. A brownish yellow is more suitable for elderly persons. A brunette should wear a green yellow. Pale green is trying to the majority of faces.

### AN OLD USE FOR HAMMERS.

"I remember," says a correspondent of the *Medical and Surgical Journal*, "that when I was very young they used to raise blisters with boiled hammers. Old Dr. Twitchell, of Keene, (peace to his ashes!) once wanted to blister some one in a farm house, far from home. He had nothing with him to do it with. He asked his wife to find him a hammer. The article was brought out, put in a teakettle over the fire, and, after the water steamed and bubbled well, he lifted it out and gently touched it to the patient, in half a dozen spots over the seat of pain, with very positive effect. Boiled hammers were for many years used in that neighborhood for pleurisy, and every old lady knew nothing was equal to a hammer; and there was a long dispute whether it should be a claw hammer or not. I think the yeas finally conquered."

### BRONZE PRINTING.

Gold dust, for printing in gold, known under the name of bronze, comes mostly from Fuerth, in Bavaria. Fine cuttings of the metal are mixed with a sticky liquid and then ground like paints. Thus reduced to a powdered form, the sticky matter is separated from the metallic dust by washing in water, after which it is sifted into the various sorts. It is obtained in as many as fifteen grades of fineness, and in the different colors—white (made from silver leaf), pale yellow, orange, green and red. In printing in gold the impressions are first struck off in printer's ink or sizing. The gold or bronze dust is then applied by means of a cotton tuft or a brush of short fur. If it is desirable to have very rich gilding, bronzes of various colors may be used. Care must be taken that the paper which is used for the bronzing process is perfectly dry.

### MONTANA HUMOR.

HERE is an instance of true politeness only too rarely met with: A Montana man dined in Philadelphia recently, and describes the event in a letter to a New York friend: "After all that fine dinner they brought in some glass bowls, with water and a piece of lemon in each. I supposed it was lemonade, and, as we had been drinking so many kinds of wine, I was glad of it, for I was very thirsty. But when I went to taste it I found it so sour that I asked for sugar. The ladies appeared to be smiling at something, but I scarcely knew what. Afterward I was told they were finger bowls to rinse one's hands in. But, John, nobody washed their hands after I drank my lemonade. I call that thoughtful, don't you?"

### FINDING THE LATITUDE AT SEA.

COMMONLY the seaman trusts to the observation of the sun to give him his latitude. The observation is made at noon, when the sun is highest above the horizon. The actual height is determined by means of the instrument called the sextant. This instrument is so devised that the observer can see two objects at once, one directly and the other after the reflection of its light; and the amount by which he has to move a certain bar carrying the reflecting arrangement, in order to bring the two objects in view in the same direction, shows him the real divergence of lines drawn from his eye to the two objects. To take the sun's altitude, then, the observer takes the sun as one object and the horizon directly below the sun as the other; he brings them into view together, and then looking at the sextant to see how much he has to move the swinging arm which carries the reflecting glasses, he learns how high the sun is. This being done at noon, with proper arrangements to insure that the greatest height then reached by the sun is observed, at once indicates the latitude of the observer. Suppose, for example, he finds the sun to be 40 degrees above the horizon, and the Nautical Almanac tells him that at the time is 10 degrees north of the celestial equator, then he knows that the celestial equator is 30 above the southern horizon. The pole of the heavens is, therefore, 60 degrees above the northern horizon, and the voyager is in 60 degrees north latitude. Of course, in all ordinary cases the number of degrees is not as exact as here for simplicity is supposed, and there are some niceties of observation which would have to be taken into account. But the principle of the method is sufficiently indicated by what has been said.

### ROUGH ON THE PARSON.

A Scotch parish once had for a minister a good man, remarkable for his benevolent disposition. Meeting one of his parishioners one day he said, "Jeanie, what way do I never see you in the kirk?" Weel, sir," said Jeanie, "to be plain wi' ye, I haena a pair of shoon to gang wi." "A pair o' shoon, Jeanie! Jeanie, I'll not let you stay at hame for that; what would a pair cost?" "About four shillings, sir." Putting his hand into his pocket he gave Jeanie the money and went his way. Some time after, meeting her again, he said, "Dear me, Jeanie, I've never seen ye in the kirk yet; what way is that?" "Weel, sir," said Jeanie, "to be plain wi' ye, when the weather's guid, and I has time, I prefer gaun to Dumfarlin, to hear Mr. Gillespie." "O, indeed, Jeanie, lass, that's the way o't, is't? You might hae gi'en me the first day o' the shoon ony way, d'ye no think?"

### YOUR LETTER.

I am going to burn your letter ; I sit by the hearth alone ;
I hear on the pane the sobbing rain, I hear the night winds moan ;
But safe in its hushed tranquility lies the little world of home,
While the firelight flashes fitfully about the quiet room ;
And brightly it glints on the checkered tints of the paper in my grasp—
Safe for a moment yet, my prize, in my loving, lingering clasp ;
Safe, though around the funeral pyre the fierce flames flash and leap,
For I shall burn your letter love, and to-night before I sleep ;

Yes, I shall burn your letter ; though again, and yet again
I read the words, as to music's chords one sets a sweet refrain ;
Though I open it where the firelight falls, with touches slow and soft,
And bend once more o'er the written words that I have traced so oft,
To please myself by fancying, "he smiled when that phrase he wrote ;"
Or, "there a shade like a summer cloud o'er the clear brown eyes would float ;"
Or, "here did he leave a kiss, like me, all for the fond word's sake,
Or for the love of the echo that he knew their melody would wake ?"

Still, I shall burn your letter ; I think I have found a joy
Whose centred pleasure nor thought can measure, nor time nor tide destroy ;
I know I wish my life may pass ere that dream dissolved I see ;
Yet I will keep our secret, love, for you, and the angels, and me ;
Our bond is hard to understand, though its weft is true and pure,
And I might die to-night, you know ; the flame is a guardian sure.
Well, flowers spring thick in summer ; my heart has a bright new June,
And I say, as I burn your letter, "He will write me another soon."

### THE WRONG FLEA.

A PERFORMANCE of educated fleas is at the present time attracting much attention in Berlin. At a recent exhibition one of the most accomplished of the insects, obeying a sudden impulse of its nature, sprung from the table and took refuge on the person of an illustrious lady. The exhibitor was in despair, as the truant was his best performer, and said he would be ruined unless it could be recovered. The lady good naturedly retired to an adjoining room, and, after a few minutes' absence, returned with the flea between her thumb and forefinger. The exhibitor took it eagerly, gave one look at it, and then, with visible embarrassment, said, "Your Highness will pardon me, but this is not the right flea!"

### WIT AND FUN.

"WHERE did you get the money with which you made the purchase spoken of?" asked the counsel?

"None of your business!" thundered the victim.

"Now, may it please your honor, are counsel to be insulted in this manner?" appealed the lawyer.

"Witness," said the Judge, compassionately, "do you wish to change your last answer?"

"No, sir, I don't."

"Well, I would not, if I were in your place!"

And the chuckle that shook the bench was audibly echoed.

JOHN PAUL says: "When a man walks squarely up to the clergyman who married him three years before, takes him by the hand cordially, and, without a word of reproach, inquires after his health, it is useless for any one to maintain that Christian forgiveness is a thing of the past."

A LOVER once wrote to a lady who rejected him, saying that he intended to retire to some "secluded spot and breathe away his life in sighs!" To which the lady replied by inquiring whether they were medium or large size. The young man has not since been heard from.

MACREADY PERPLEXED.—When Macready, the actor, visited this country he found many things to puzzle and perplex him, for he was as precise and angular as Grewgious. The idioms and the eccentricities of the Yankees were beyond his comprehension. At one of our theatres, where he was performing an engagement, he had occasion to find fault with the supporting actors, who were a particularly free-and-easy set. Going to the manager one day, he said:

"Mr. Manager, you have deceived me, sir. You have told me that which was not true, sir!"

"Bless me!" cried the manager, in surprise, "how so?"

"About your actors, sir. Did you not tell me that Mr. A—— was on a high?"

"Yes."

"And that Mr. S—— had a touch of the tanglefoot?"

"Yes."

"And that Mr. P—— had a brick in his hat?"

"Aye—that was what I said."

"And, in explanation of the conduct of Mr. B——, you told me that he had a snake in his boot?"

"Certainly I did."

"Well, sir," announced the great tragedian, in his most stern and indignant manner, "I find, upon critical examination, that these men are all drunk, sir! Aye—all drunk!"

Too Refined for Texas Society.—A few days ago a wagon, drawn by a yoke of long horned Texas cattle, halted in Jackson, Minn. The wagon contained a good looking woman, seven children, and considerable plunder. A man, a small boy, and a dog that had run to tail, were the adjuncts. The party were from Texas, and returning to their old home in Decatur County. An alderman of this city, who had passed many years of his life in the Lone Star State, approached the wagon. He said to the woman, "From Texas, I presume?" "Yes, sir." "Didn't you like the country?" "No, sir." "Didn't you like the climate?" "Oh, yes." "Did you have good health out there?" "Yes." "Wasn't the land good?" "Yes." "How about the crops?" "Oh, we made splendid crops." "Well, then, ma'am, what on earth is your objection to Texas?" "Why, sir," she replied, "I couldn't stand the society in that rough country;" and then she turned to the small boy, her son, and cried, "Sam, drive that d—d dog out'en the dinner pot; don't you see he's got his nasty snout into the vittils?"

Now here is a case of pure innocence. An Iowa railroad employé, whose signal lantern, like that of the foolish virgins, was left without oil, wrote to the supply officer for "some more of that red oil," not knowing that the color of the lantern globe had something to do with the shade of light it threw out.

A member of the Ohio Legislature recently visited the State Penitentiary, and while there had his hair cut by the prison barber. While undergoing the tonsorial process a party of lady and gentlemen visitors looked in, and the legislator was made happy by catching such remarks as, "Ugly look about the eyes," "Vicious mouth," "Looks capable of any wickedness," "Wonder what he did?" etc.

A Cannon Ball in the Hat.—An anonymous writer, generally supposed to be Rev. Henry Ward Beecher, after describing how, when a boy, he stole a cannon ball from the navy yard at Charlestown, Mass., and with much trepidation, and more headache, carried it away in that universal pocket of youth, his hat, winds up with the following reflections—reflections which, though philosophically trite, are in this manner conveyed with much force and freshness:

"When I reached home I had nothing to do with my shot. I did not dare to show it in the house, nor tell where I got it, and after one or two solitary rolls I gave it away on the same day to a Prince streeter.

"But, after all, that six pounder rolled a good deal of sense into my skull. I think it was the last thing that I ever stole (excepting a little matter of heart, now and then), and it gave me a notion of the folly of coveting more than you can enjoy, which has made my whole life happier. It was rather a severe mode of catechizing, but ethics rubbed in with a six pounder shot are better than none at all.

"But I see men doing the same thing—going into underground and dirty vaults, and gathering up wealth, which will, when got, roll around their heads like a ball, and be not a whit softer because it is gold, instead of iron, though there is not another man in Wall street who will believe that.

"I have seen a man put himself to every humiliation to win a proud woman who had been born above him, and when he got her he walked all the rest of his life with a cannon ball in his hat.

"I have seen young men enrich themselves by pleasure in the same way, sparing no pains, and scrupling at no sacrifice of principle, for the sake at last of carrying a burden which no man can bear.

"All the world are busy in striving for things that give little pleasure and bring much care. I am accustomed, in all my walks among men, noticing their folly, to think, 'There's a man stealing a cannon ball;' or, 'There's a man with a ball on his head; I know it by his walk.'

"The money which a clerk purloins for his pocket at last gets into his hat like a cannon ball. Pride, bad temper, selfishness, evil passions will roll a man as if he had a ball on his head! And ten thousand men in New York will die this year, and as each one falls his hat will come off, and out will roll an iron ball, which for years he has worn out his strength carrying."

A Sell—Not a Sale.—Amiable shopkeepers deserve to be canonized. Here is an illustration of the trials to which they are constantly subjected. One midsummer day, when Æolus slept, and the thermometer stood in the nineties, a lady entered a store not a thousand miles off and inquired for parasols. The obliging proprietor spread out before her samples of a large and varied stock. "Have you any of this shade a size larger?" said the lady. The size larger was produced. "I think, on the whole, I prefer the size smaller." The size smaller was presented. "Have you any of this size a lighter shade of blue?" The required shade was brought out. "Haven't you any of this kind with a crooked handle?" The shade with the crooked handle appeared. "Have you any with the crooked handle not quite so heavy?" said the lady, and so continued her inquiries for every conceivable size, shade and weight possible in the line of parasols. After nearly an hour had been thus consumed, the fair shopper gathered up her handkerchief and gloves and moved for the door. "Can't I sell you a parasol?" inquired the exhausted proprietor. "O dear, no," replied the lady, "I was merely inquiring the prices. I am going into mourning myself, and have one for sale."—*New Bedford Mercury.*

WHY BARNEY WAS RETAINED.—A firm dealing largely in coal in one of our Western cities had in their service an Irishman named Barney. One day the head of the firm, irritated beyond endurance at one of Barney's blunders, told him to go to the office and get his pay, and added: "You are so thick headed I can't teach you anything." "Begorra," says Barney, "I larnt wan thing since I've been wid ye!" "What's that?" asked his employer. "That sivinteen hundred made a ton." Barney was retained—or, to use the phraseology of a Southern gentlemen who has just won the heart and hand of one of New York's most opulent widows, "he resumed the primeval condition of his former rectitude."—*Editor's Drawer, in Harper's Magazine for April.*

### STORY OF AN INKSTAND,

### WITH THE INKSTAND LEFT OUT.

"THAT inkstand? Yes, it has a history. I may as well tell it to you now as any other time, I suppose. You ought to be called the Great American History Extractor, or Romance Extractor; for, if there is a particle of romance in anything or anybody in a place where you happen to be, you are sure to scent it out.

"Of all days this is the most glorious one for a romance—the rain falling with that steady, monotonous drip, drip, drip; not a soul in the house but ourselves, and we so snug in this splendid old library."

Mag Hastings was, indeed, an indefatigable romance hunter. She was always looking for situations where the romantic predominated. She would have succeeded as a dramatist, without a doubt. She was apparently as happy as mortal could be on the morning in question, nestled in the depths of my scarlet lounge, perfectly certain that she looked picturesque in her dark green street suit, relieved by the daintiest linen.

"Come here, pet!" said she; and a bunch of white wool, with a blue ribbon tied at one end of it, marking a spot where in a dog a neck would be, trotted mysteriously toward her. What propelling power there could be in the shaggy thing was a wonder I could never get over; but, somehow, it managed to spring into Mag's arms, and then the silence assured me that my story was expected.

The surroundings were favorable for story telling, it is true. The room heavily wainscoted with dark wood; the cases of books of all times and of all varieties; the long windows richly draped with scarlet brocade, lined with exquisite lace; the thick soft carpet of mottled green; the Turkish lounges, the quaint chairs, luxuriously upholstered; the bronzes on the mantle and in niches and corners—bronzes that told old stories of mythology; the engravings on the wall; the little gems in oil selected for their wonderful coloring—an autumn scene, a burning ship, a group of German peasants; the glowing fire of sea coal, and the polished hearth and fender—all appealed to the love of the beautiful and picturesque, while the cold, unceasing rain, the bare, guant trees, the dripping shrubbery, and the blanched grass, drove the thoughts within for solace and amusement.

The inkstand that Mag Hasting referred to was a pretty affair in Swiss carving. It represented two little peasants carrying water, the buckets suspended from a pole resting in a hand of each. Of course, the water pails were the ink receptacles, and the pole was the pen rack. It was poetic and realistic at the same time, and as pretty a trifle for a library table as one would wish to see.

"The story is a sad one, Mag," said I. "It will give you the blues for the rest of the day."

"So much the better," she answered, with a true dramatic love of the horrible—"so much the better. I'd like to be stirred up a little. I fear I'm too comfortable. A little dash of imaginative sorrow is needed to relieve this perfect enjoyment. A little shade throws out the good points of anything, you know. Isn't it so, pet?" and she pinched the little appendage dignified by the name of tail till a sharp yelp came out of the soft white mass in her arms. "There, I told you so, pet; now you'll know what true repose is."

"I should scold you, Mag," said I, "if were the least use in the world. But you are incorrigible; so I will go on. Time will give you shadow enough, without doubt."

"When I was in Venice—" I commenced.

"In Venice!" exclaimed Mag, sitting bolt upright, and giving the little dumpling of a poodle a push that brought out a most spiteful yelp. "When were *you* ever in Venice?"

"Why, you know, Mag, that I was abroad more than two two years. In fact, I had just returned when you and I became acquainted," I replied, wondering a little at the unusual interest she appeared to take in the commencement of my story.

"Oh, I knew that," she replied. "But I never heard you say anything about Venice;" again reclining and closing her eyes, as much as to say, "Go on. I am at a loss to conçeive what made me so foolish as to disturb myself for so slight a thing as the mention of a foreign and defunct city."

"Well, when I was in Venice—I believe it was the second month of my stay there—Charles came in one day (Charles is my husband) from a long tramp about the Palazzo Loredan, the Ca' d' Oro—you know which I mean—the one built in the sixteenth century, in the Oriental style, and restored by Mademoiselle Taglioni, the celebrated dancer."

"Oh, yes," said Mag, impatiently, "I know all about it. Haven't I been here? wasn't I born there? haven't I always lived there? didn't the Doge of Venice christen me? didn't I draw my first breath on the Bridge of Sighs? and wasn't I ore one of Mademoiselle Taglioni's pupils? Go ahead, and tell what Charles said when he came back from his tramp about the Ca' d' Oro. But never mind the architecture; I'm not building at present."

I had seen Margaret in many strange moods, but never felt her to be so utterly incorrigible as upon this occasion. However, I resumed without appearing to remark it.

"Charles came in, and said, hastily, 'Nell, tell Pedro to get up a nice lunch just as quick as he can. I have brought an invalid home with me, and, if I can, I shall persuade him to remain a while with us. I have taken a strange fancy to the fellow, and should like to have him where we could take a little care of him. He will certainly die if somebody don't take an interest in him.' So I hurried around, and after a little Pedro and I—"

"Oh never mind about the lunch!" interrupted Mag again, without opening her eyes, and with a little tremolo in her voice which I could not understand. "Proceed with the man! Animals always interest me more than food."

"You would not call Austin Benedict an animal if you could see him once," I replied, a little indignantly, and was about to add that I didn't wish Charles mixed up with that species, either, when over went the lap dog on to the lounge, and Mag said, irritably:

"I believe *that* animal thinks I have nothing to do but to make my lap into a bed for his convenience. Go on, Nell. Austin Benedict! Austin Benedict is a good name. It has got the right kind of ring to it. I'll bet my new solitaire against three cents that his character was as stony as his cognomon. A man called Austin Benedict would do what he considered to be right, if by so doing it killed him and everybody he was acquainted with. I wish you would ring for some wine, Nell, I am as cold as death. Don't get up, though—and please go on."

"Yes," I resumed, "you are quite right about Mr. Benedict's character. I believe there is something in names. But, for all that, the fellow was dying for love."

"A very interesting case," said Mag, turning deathly pale. "Do you know the circumstances?" and then, with the slightest perceptible sneer, added, "A man must be very strong to admit such a thing about himself."

"Oh, I answered, "it was a long time before we got at the facts in the case; but one day, when I sat by him, and we all thought he couldn't last many hours, he told me the whole story."

"When you sat by him, and thought he couldn't last many hours, he told you the whole story?" repeated Mag, in a strange sort of a way. "Did he *die?*"

"No; he rallied again," I answered, almost out of breath at Mag's strange behavior. "It seems that he loved with his whole heart and soul a very beautiful and much sought after young lady. She *pretended* to love him. He parents were opposed to the match; she proposed to defy her parents. The next news he receives comes in the form of a letter from her, telling him that she finds she does not care for him as she supposed, and asking to be freed from her engagement."

"He *did* receive such a letter, did he? Austin Benedict did receive such a letter?" and Mag arose from her recumbent position, and stood before me, pale as a corpse, but with the light of forty avenging angels in her eyes. "I never wrote that letter!"

she exclaimed. "It is a forgery from beginning to end! Nell Harris, you took care of Austin Benedict in his last hours?" And now the proud head of Margaret Hastings was buried in my lap.

"I took care of him when he was ill," I replied.

"And he loved the woman he believed to be false to the last?"

"He loved the woman—"

"Oh, Nell! Nell! what shall I do? How can I ever live, now that I know he died with that cruel impression of me?" interrupted poor Mag, giving me no opportuity to explain myself. "You took care of him—you made him comfortable—you kissed him when he was dying; and I—loved and despised, I—"

It was about time for me to insist upon being listened to; so I said, "Stop a minute, Mag, darling. I did do all I could for Austin Benedict's comfort, and have kissed him a good many times, but not when he was dying, Mag, for he hasn't passed away yet, unless he has accomplished that feat to-day, and—and—"

"What in the world is all this?" said Charles, who had entered softly with his latch key.

"Where is Austin?" I asked in a whisper; for Mag was so still I didn't know but that she was dead.

"Here," said the dear fellow, bounding forward. He stopped suddenly at sight of the figure at my feet. "In the name of the angels! Nell, what is this? and whom have you got there?"

One little faint cry from Mag, and she was a dead weight in Austin Benedict's arms. That was answer enough.

Such a day as that was! Between swoons, explanations and embraces, my mind got to running upon lunatic asylums; but the sun set clear, and my reason remained unobscured. There was a wedding that same evening in the same library; and, in all the happiness I ever witnessed—Charles's and mine thrown in—I know I never saw such perfect, unalloyed joy as exists between Mr. and Mrs. Austin Benedict. Mag didn't give me time to get to the inkstand, so you must imagine the history of that.

